# Variants of *IFNL4* Gene in Amazonian and Northern Brazilian Populations

**DOI:** 10.3390/genes14112075

**Published:** 2023-11-14

**Authors:** Carolina Cabral Angelim, Letícia Dias Martins, Álesson Adam Fonseca Andrade, Fabiano Cordeiro Moreira, João Farias Guerreiro, Paulo Pimentel de Assumpção, Sidney Emanuel Batista dos Santos, Greice de Lemos Cardoso Costa

**Affiliations:** 1 Programa de Pós-Graduação em Agentes Infecciosos e Parasitários, Laboratório de Genética Humana e Médica, Federal University of Pará, Belém 66073-000, PA, Brazil; carolina.angelim@icb.ufpa.br (C.C.A.); lemartias@hotmail.com (L.D.M.); alesson.andrade@icb.ufpa.br (Á.A.F.A.); sidneysantos@ufpa.br (S.E.B.d.S.); 2 Programa de Pós-Graduação em Virologia, Instituto Evandro Chagas, Ananindeua 67030-000, PA, Brazil; 3 Programa de Pós-Graduação em Genética e Biologia Molecular, Laboratório de Genética Humana e Médica, Federal University of Pará, Belém 66073-000, PA, Brazil; fcmoreira@ufpa.br (F.C.M.); joaofg@ufpa.br (J.F.G.); 4 Núcleo de Pesquisa em Oncologia, Federal University of Pará, Belém 66073-000, PA, Brazil; assumpcaopp@gmail.com

**Keywords:** IFNL4, Amazon population, mixed population, Brazil, genetic variants

## Abstract

Since the discovery of the polymorphic nature of the *IFNL4* gene, its variants have been investigated and associated with several viral diseases, with an emphasis on hepatitis C. However, the impacts of these variants on mixed-race and native populations in the northern region of Brazil are scarce. We investigated three variants of the *IFNL4* gene in populations from this location, which were among the 14 most frequent variants in worldwide populations, and compared the frequencies obtained to populational data from the 1000 Genomes Project, gnomAD and ABraOM databases. Our results demonstrate that mixed-race and native populations from the northern region of Brazil present frequencies like those of European and Asian groups for the rs74597329 and rs11322783 variants, and like all populations presented for the rs4803221 variant. These data reinforce the role of world populations in shaping the genetic profile of Brazilian populations, indicate patterns of illness according to the expressed genotype, and infer an individual predisposition to certain diseases.

## 1. Introduction

The study of genetic variants has acquired significant importance in recent years, as it provides explanations for different clinical conditions and a profile for responses to therapeutic treatments among individuals [1]. In infectious and contagious diseases, several genes have been investigated, and especially in viral infections, the genes that encode interferons deserve to be highlighted because their translation generates proteins linked to antiviral responses [2].

Type III interferons—the family of lambda interferons (*IFNL*)—are considered specialists in antiviral action, blocking stages of the viral replicative cycle, increasing antigenic presentation, and triggering antiproliferative properties [3]. The notoriety of type III interferons began in 2003 with the description of IFN lambda 1 (*IFNL1*), lambda 2 (*IFNL2*) and lambda 3 (*IFNL3*). After 10 years of studies, a fourth type was identified, called IFN lambda 4 (*IFNL4*) [4]. This one stands out from the others due to its highly polymorphic character, followed by an apparent relationship with the sustained virological response (SVR) and the clearance of some viruses, justifying its importance.

The expression of IFN-λ4 occurs in several cell types, but its recognition is limited to cells that express the IFNLR1 receptor [3,5]. Studies suggest that this cytokine may have evolved specifically to protect the epithelium, as its antiviral effects may have begun before IFN-λ1, IFN-λ2 and IFN-λ3 [6]. The signaling initiated by IFN-λ4 activates the JAK-STAT pathway, leading to the expression of interferon-stimulated genes (ISGs) such as Oligoadenylate Synthetase 1 (OAS1) and Ubiquitin-specific Peptidase 18 (USP18) [1].

Studies on type III interferons emphasize how these cytokines have potential in vitro and in vivo against many viruses, such as dengue virus (DENV), cytomegalovirus (CMV), human immunodeficiency virus (HIV), and Epstein–Barr virus (EBV) [1,7]. However, the greatest potential of this gene resides in associative studies of *IFNL4* variants and the spontaneous clearance of the Hepatitis C virus (HCV), with effects on therapies against this infection [8,9].

Despite the time that has passed since the discovery and description of this gene until today, there are still few studies on the impact of its variants on world populations. Proof of this are clinical data from banks such as ClinVar—National Center for Biotechnology Information; BraVE, LovD, Beacon—Brazilian Initiative on Precision Medicine—that need to be updated [10].

The study of the distribution and prevalence of genetic variants of certain genes in mixed populations is particularly important. They can indicate patterns of illness according to the expressed genotype and infer an individual’s predisposition to certain diseases [11,12,13]. It is important to focus on viral hepatitis which, according to the World Health Organization, varies in prevalence across continents due to the mode of transmission, existing viral genotypes, available prophylactic and therapeutic measures [14,15].

We add here, as a factor in this variability, the heterogeneous genotype distribution of genes such as *IFNL4*, especially in infections by hepatitis B (HBV) and C (HCV) viruses [16,17,18]. These diseases have a high distribution in African countries, East and Southeast Asia and European countries, and we observed that the populations of these countries have varying genotypes for the *IFNL4* gene [19,20,21].

This variability is often caused by a single allele, which can condition a better or worse resolution of viral infections and therapeutic responses [22,23,24]. As an example, we have the rs12979860-C/T variant in which the allelic replacement of T by C conditioned sustained virological response (SVR) in European and Asian populations, in addition to a better response to treatments with sofosbuvir [25,26,27].

In this sense, it is understood that investigations of genetic variants such as single nucleotide polymorphisms (SNPs) and insertion or deletion (INDEL) allow the discovery and characterization of new molecular markers, with clinical applications functioning in prognosis or even in therapeutic decisions for some population groups.

Therefore, our study aimed to characterize the molecular profile of the *IFNL4* gene in a mixed and native population from the Brazilian Amazon and compare these findings with data from the general Brazilian population described in the Online Archive of Brazilian Mutations (ABraOM), and world populations available in the 1000 Genomes Project and Genome Aggregation Database (gnomAD).

## 2. Materials and Methods

### 2.1. Identification of Variants

The first stage of this research was an exploratory search in the EnsEMBL (https://www.ensembl.org/index.html, accessed on 21 April 2023) database, which uses data from the 1000 Genomes Project, and GnomAD (https://gnomad.broadinstitute.org/, accessed on 22 April 2023). In these databases, we admitted the Minor Allele Frequency (MAF) on the most frequent variants (MAF > 0.05) of the IFNL4 gene and these were investigated in world populations that could serve as a reference for Brazilian populations, especially Native Americans and mixed-race people from the Northern region of Brazil.

For comparisons between the database samples and the study-elected populations, we are only considering the exon variants of this gene since we aim to investigate exome data from mixed-race and native Amazonian populations. For specifics about the Brazilian population, the source database was ABraOM (https://abraom.ib.usp.br, accessed on 22 April 2023).

### 2.2. Data Analysis

This section of the study was carried out in two different ways, depending on the data bank considered. For EnsEMBL, accepting that the information available stems from 1000genomes and GnomAD, carrying a worldwide perspective, the steps were laid out as: (I) Aiming for an accurate investigation and comparison of variants that were found in Amazonian populations, the data acquisition phase was designed to include Minor Allele Frequencies (MAF) 5%; (II) using the search query “IFNL4” and selecting the matching result (gene symbol report and Hugo Gene Nomenclature Committee (HGNC) ID: 44480); (III) choosing “variant table” and adjusting the MAF upwards, so the results would depict the percentages from highest to lowest; (IV) organizing a primary table on Google Docs with columns detailing populations in order of: variant (rs), all (global percentages), AFR (African Population), AMR (American Population), EAS (East Asian Population), EUR (European Population), SAS (South Asian Population); (V) from the data generated, a second organization was made on Google Spreadsheets detailing the following: chromosome, position, reference nucleotide, variant nucleotide, amino acid and protein changes, variant kind and allelic information—number, count, frequency, homozygous or heterozygous. The study condensed these and added information from our native population as well.

When considering ABraOM, we had a more local approach since this data bank only considers data generated from Brazilian populations (Data Collection from São Paulo, SP, Brazil). For this database, the steps were: (I) opening the website, selecting SA-BE-WGS-1171 (hg38) and searching for “IFNL4”; (II) selecting the highest frequency option for the variant order display; (III) exporting the data to a Google spreadsheet considering: chromosome, position, reference and variant nucleotides, category, consequence predictor (if any, using tiers of intron, exon, substitutions or unknown) rs ID and their population frequencies (same population order as the worldwide banks, with the rs information also being crossed with NCBI references), homozygous and heterozygous numbers, allele number, count and frequency were sorted highest to lowest. All data were collected and organized between April and July 2023.

### 2.3. Study Populations

**Figure 1:**
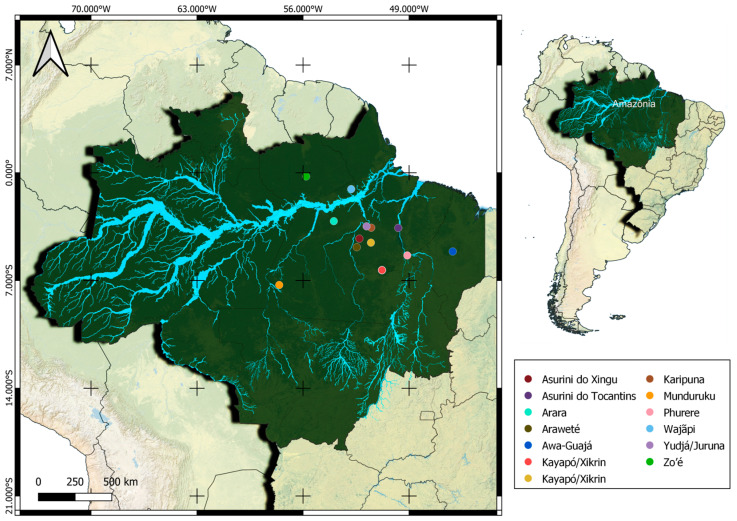
Location of Amazonian tribes present in this study. Image made on QGIS 3.28.0 software.

This study uses population data described by Cohen-Paes and collaborators in 2022 [28], which includes 63 Indigenous people, representatives of the 12 Amazonian ethnicities from the northern region of Brazil. They are from Asurini do Xingu, Arara, Araweté, Asurini do Tocantins, Awa-Guajá, Kayapó/Xikrin, Zo’é, Wajãpi, Karipuna, Phurere, Munduruku and Yudjá/Juruna tribes and their ancestry was confirmed by their exomes. Table 1 summarizes demographical information about these groups and Figure 1 presents their location in Brazil.

For our comparative analysis, the Indigenous groups were called Native American Population (NAT). Furthermore, this study includes 89 mixed-race individuals from the metropolitan region of Belém (PA), which includes the cities of Belém, Ananindeua, Marituba, Benevides, Santa Barbara, Santa Izabel, Castanhal and Barcarena; referred in this work by the acronym MISC.

The study was approved by the National Research Ethics Committee and the Research Ethics Committee of the Tropical Medicine Center of the Federal University of Pará (CAE: 20654313.6.0000.5172). The participation of all individuals was conditioned on acceptance and signing of the informed consent form (ICF).

The data were compared to samples present in the databases 1000 Genomes (http://www.1000genomes.org, accessed on 19 April 2023) and GnomAD (https://gnomad.broadinstitute.org/, accessed on 19 April 2023), which are organized by presenting five continental populations including African (AFR), East Asian (EAS), South Asian (SAS), European (EUR), and American (AMR) individuals. Comparisons to the Brazilian population were obtained from the ABraOM database (https://abraom.ib.usp.br/, accessed on 19 April 2023).

### 2.4. DNA Extraction and Exome Library Preparation

DNA extraction occurred using the phenol-chloroform method [30]. DNA quality was assessed using the Nanodrop-8000 spectrophotometer (Thermo Fisher Scientific Inc., Wilmington, DE, USA). Library preparation used the Nextera Rapid Capture Exome (Illumina^®^, SanDiego, CA, USA) and SureSelect Human All Exon V6 (Agilent technologies, Santa Clara, CA, USA), following the manufacturer’s recommendations. Sequencing took place on the NextSeq 500^®^ platform (Illumina^®^, San Diego, CA, USA) with use of the NextSeq 500 High-output v2 300-cycle Kit Kit (Illumina^®^, San Diego, CA, USA).

### 2.5. Bioinformatics Analysis

The resulting reads in FASTQ format were analyzed (FastQC v.0.11 http://www.bioinformatics.babraham.ac.uk/projects/fastqc/, accessed on 25 June 2023) and low-quality reads were eliminated (fastx_tools v.0.13 http://hannonlab.cshl.edu/fastx_toolkit/, accessed on 25 June 2023). The sequences were aligned with the reference genome (GRCh38) using the BWA v.0.7 tool (http://bio-bwa.sourceforge.net/, accessed on 25 June 2023).

The sequencing reads were cut to Illumina adapters and filtered using Trimmomatic v.0.36 [31] the remaining reads were aligned to the hg19 human genome reference, using BWA MEM v0.7 [32,33]. The PCR duplicates were removed via Samblaster v.0.1 [34] and the resulting mapped reads were classified and indexed making use of Samtools v1.8 [35] and Sambamba v.0.6 [36]. Lastly, the quality of the mapped bases was assessed and recalibrated with GATK v.4.0.0 BaseRecalibrator and ApplyBQSR.

Allelic variants were noted in ViVa^®^ (Viewer of Variants, Natal, RN, Brazil). Markers in the IFNL4 gene were selected from the generated coverage (fastx_tools v.0.13-http://hannonlab.cshl.edu/fastx_toolkit (accessed on 25 June 2023)).

### 2.6. Statistical Analysis

The allelic frequencies were compared to verify if the mixed-race (MISC) and native (NAT) Amazonian populations present some sort of Allelic Distribution of the *IFNL4* gene variants, similar to those observed in Continental and Brazilian populational groups. For that, we used the Fisher’s exact test. The results were considered statistically significant when the *p* value was less than 0.05 (*p* ≤ 0.05). The Benjamini-Hochberg False Discovery Rate (FDR) [37] test was used as a statistic correction measure applied to all the multiple tests listed. All statistical analyses were performed using the R Studio v.3.5.1 program (R Foundation for Statistical Computing, Vienna, Austria).

The LD Link tool [38] was employed to extract data referring to variant linkage disequilibrium from the ones found both in this study and in public databases, such as the 1000 Genomes Project. The results are shown through a media estimate based on the proximity between two variants (D’) in the investigated populations.

## 3. Results

In exploratory research, we identified 10 variants in which the altered allele was frequent in at least 10% of the populations described by the 1000 Genomes Project (MAF > 10%) and another four variants with a frequency of 5% (MAF > 5%). Among them, two variants identified in mixed-race and native populations in the northern region of Brazil had a frequency greater than 10%, and one had a frequency greater than 5%. Table 2 summarizes the characteristics of the collected variants, namely: location of the variant on the chromosome, its identification, altered alleles, variant type, and its consequence.

Exome analysis of samples from mixed-race and native Amazonians showed three of these variants being 2 SNVs (single nucleotide variant) and 1 INDEL (Insertion-Deletion). The SNV variants were rs74597329-G/T, rs4803221-C/G and are classified as moderate impact and the INDEL type variant –rs11322783-ΔT/TT–of high impact as it is a frameshift mutation.

Table 3 below presents the allelic frequencies of the *IFNL4* gene variants in each of the five population groups described by GnomAD v3.1.2—Africa, Americas, East Asia, Europe, and South Asia, while Table 4 presents the frequencies observed in the 1000 Genomes Project in the same groups—the frequencies are presented in decimal number format, in both tables. They also contain the frequencies of São Paulo population obtained from the ABraOM database, serving as a basis for comparison of the frequencies observed in mixed-race and Native American populations in the Northern region of Brazil.

We observed subtle differences between data from GnomAD and the 1000 Genomes Project (Table 5 and Table 6). Pairwise comparison with data from the 1000 Genomes Project showed that the native group (NAT) differs from AFR (*p*-value: <0.0001), AMR (*p*-value: 0.0097), and São Paulo—AbraOm (*p*-value: 0.0097) in rs74597329 and rs11322783 variants. In the rs4803221 variant, NAT was also different from AFR (*p*-value: 0.0164) and AMR (*p*-value: 0.0031) groups. The mixed group (MISC) presented differences when compared to AFR (*p*-value: <0.0001) and EAS (*p*-value: 0.0031) for rs74597329 and rs11322783 variants. value: 0.0031) groups. The mixed group (MISC) presented differences when compared to AFR (*p*-value: <0.0001) and EAS (*p*-value: 0.0031) for rs74597329 and rs11322783 variants.

Table 6 refers to pairwise comparisons between our local data and data from GnomAD. It also shows differences between NAT, AFR, AMR and Southern Brazilian population in rs74597329 and rs11322783 variants. AMR and AFR groups also differ from NAT for rs4803221 variant (*p*-value: 0.016 and 0.003). Moreover, examining the mixed population, we saw that either AFR (*p*-value < 0.0001) and EAS (*p*-value = 0.0003) differs from it for rs74597329 and rs11322783 variants. The differences can also be noticed in the plot of Supplementary Materials S1. The analysis of connection imbalance between the variants observed in the present study shows a strong imbalance of these variants (r^2^ = 0.990; D’ = 1) in all populations already observed and, therefore, they can be analyzed as a haplotype [38].

## 4. Discussion

Modern-day Latin American people are the result of a complex process of mixing different genetic profiles [39]. The studies on genetics are still scarce, especially on Amazonian natives, which makes it difficult to know the specifics of their singular profile in comparison to other groups. Many authors highlight that allelic variance in genes present amongst different ethnic groups may show associations with significant consequences for human illnesses [13,40,41,42].

This can be clearly exemplified by the *IFNL4* gene, whose variants may regulate IFN-λ4 expression and functionality. Interferon lambda proteins can activate the JAK-STAT pathway, leading to a positive expression of genes with antiviral properties aiming to eliminate pathogens, such as hepatitis C virus. This gene presents interesting characteristics due to a polymorphism that regulates IFN-λ4 expression; it is composed of the rs74597329 (IFNL4-G/T) and rs11322783 (IFNL4-∆/T) variants, which are in complete connection imbalance and, due to this, are analyzed together [5,10,28].

Those variants behave as a dinucleotide variation, named rs368234815 (ΔG/TT). In practical terms, this means that individuals will always have the IFNL4-ΔG (rs11322783∆/rs74597329G) and IFNL4-TT (rs11322783T/rs74597329T) haplotypes [5,28]. IFNL4-ΔG haplotype allows the formation and expression of fully functional IFN-λ4 (p179) and the IFNL4-TT haplotype alters the reading frame of the protein, creating inactive forms (p124 and p143) [10]. A paradoxical fact is discussed in the Fang [6] and Vergara [43] studies, where they show that individuals not expressing IFN-λ4 (haplotype IFNL4-TT) have higher clearance rates and better results from treatments against HCV infection.

Our study shows that the IFNL4-TT haplotype is predominant in Amazonian natives as well as in mixed and Asian populations, reaching values between 74% and 93%. The mixed group presents a profile similar to American (AMR), European (EUR), Southern Asian (SAS) and São Paulo (ABraOm) groups. It was an expected aspect since the mixed population of the study was formed by founding groups such as Europeans and Asians [44].

The differences observed when comparing the frequencies of African people to our populations of study happen because the AFR group has a differing prevalence of the ∆G haplotype when compared to all the other populations. Some authors discuss how this strong selective pressure for the IFNL4-TT haplotype may have arisen before the migration of human populations out of Africa. Due to other factors still unknown, IFNL4-TT became stronger in European and Asian groups but not in the original population. In other words, the downregulation of this protein is beneficial in many populations. [6,45]

A proposal to justify different results in American populations is given by [46]. He proposes that the genetic variations of the native populations of North, Central and South America are different due to the colonialism process and that the South American natives are even more genetically isolated from other American populations. Therefore, frequencies observed in Brazilian population groups are the result of different formation processes among our people [47].

The variant rs4803221 has controversial results. While [48,49] show G, the minor allele, as a poor predictor of viral clearance in individuals undergoing therapy with Pegylated Interferon-α (pegIFNα), and DAAs [43] discuss that this allelic variance did not impact the elimination or remaining of viral infection in Africans.

In our study, the rs4803221 variant did not show any statistically differing results on the comparison between the NAT and MISC samples and the database-checked populations. Low frequencies of minor allele (G) were observed on the NAT and EAS samples, which is expected when we evaluate the formation process of American populations, which considers American ancestry to have come from the migration of Asians by the Bering Strait [50]. However, the frequencies of the minor allele of this variant in the MISC sample are similar to those of the groups that formed it: AFR, AMR and EUR.

The SNPs rs73555604 and rs117648444 collected in the exome of the *IFNL4* gene are shown in worldwide populations (GnomAd and 1000 Genomes) and in southeastern Brazil (ABraOm), but they are not present in the samples of our study. They are missense-type SNVs, and their absence in the sample of mixed-race and native Amazonians is explained by their minor frequencies of MAF in most continental populations, except for the AFR population, in which the frequencies of the lowest allele are slightly larger.

A better understanding of the genetic profile of our population is important to validate the existing hypotheses of distinct patterns in disease responses, infections, and treatments in different people. The importance of characterizing the genetics of native populations and people who have suffered intense processes of ethnic mixing is justified by essentially helping to understand illness processes that are, sometimes, unclear.

Other studies follow ours to elucidate (1) the distribution of these variants in patients with hepatitis C and (2) the direct consequences of these polymorphisms on both the HCV clearance and the efficiency of drug treatment in populations from the north of Brazil. This information will contribute knowledge and base the development of public policies aiming for health improvement in many groups across the country.

## 5. Conclusions

The results indicate that the variants rs74597329, rs11322783 and rs4803221 of the *IFNL4* gene evaluated in mixed-race and native populations from the northern region of Brazil have frequencies resembling those in world populations, especially Asian and European ones. In practical terms, these results suggest that world populations played a significant role in the formation of the Brazilian population. Therefore, this study aid further future public policies associated with the treatment of viral infections in these populational groups.

## Figures and Tables

**Table 1:** Populational groups demographics including the tribes, their location, number of habitants and data source.

Tribe	Location	State	Population (N)	Source
Asurini do Xingu	Altamira, Senador José Porfírio	Pará	182	SIASI
Arara/Arara do Iriri	Altamira, Brasil novo, Medicilância, Uruará	Pará	298	SIASI
Araweté	Altamira, São Félix do Xingu e Senador José Porfírio	Pará	467	SIASI
Asurini do Tocantins	Baião e Tucuruí	Pará	565	SIASI
Awa-Guajá	Arariboia	Maranhão	520	[29]
Kayapó/Xikrin	IL Bacajá (Altamira, Anapu, São Félix do Xingu, Senador José Porfírio	Pará	746	FUNAI
	IL Xikrin do Cateté (Água Azu do Norte, Marabá, Parauapebas)	Pará	1183	SIASI
Zo’é	Óbidos	Pará	330	IEPE
Wajãpi	Laranjal do Jari, Mazagão, Pedra Branca do Amapari	Pará	1454	DSEI/AP
Karipuna	TI Galibe (Oiapoque)	Amapá	151	SIASI
	TI Juminá (Oiapoque)	Amapá	291	IEPE
	Uaçá I e II (Oiapoque)	Amapá	4462	FUNAI
Phurere	Marabá	Pará		
Munduruku	Itaituba e Jacareacanga	Pará	6518	SIASI
Yudjá/Juruna	Vitória do Xingu	Pará	95	SIASI

Acronyms: IL—Indigenous land, SIASI—Indigenous Health Care Information System, FUNAI—National Foundation of Indigenous Peoples, DSEI—Special Indigenous Health Districts, IEPE—Special Indigenous Health Districts.

**Table 2:** Characterization of variants of the IFNL4 gene investigated in distinct groups: African, American, East Asian, European, South Asian, Brazilian from São Paulo, mixed-race, and Native American populations from the Northern region of Brazil.

Genomic Position	SNP Identifier	Nucleotide	Variant	Consequence
19:39246873	rs12979731	T > C	SNV	3′UTR variant
19:39246936	rs370209610	T > C	SNV	3′UTR variant
19:39246970	rs11882871	G > A	SNV	3′UTR variant
19:39247226	rs12971396	C > G	SNV	3′UTR variant
19:39247247	rs137902769	A > T	SNV	3′UTR variant
19:39247389	rs73555604	C > T	SNV	missense variant
19:39247677	rs111531283	C > A	SNV	intron variant
19:39247938	rs117648444	G > A	SNV	missense variant
19:39248147	rs12979860	T > C	SNV	intron variant
19:39248489	rs4803221	C > G	SNV	missense variant
19:39248514	rs11322783	T > TT	INDEL	frameshift variant
19:39248515	rs74597329	G > T	SNV	missense variant
19:39248713	rs4803222	G > C	SNV	5′UTR variant
19:39737578	rs570739705	C > G	SNV	3′UTR variant

**Table 3:** Frequencies of *IFNL4* gene variants investigated in African (AFR), American (AMR), East Asian (EAS), European (EUR), South Asian (SAS) from GnomAD, Brazilian population from Online Archive of Brazilian Mutations (ABraOm), mixed-race (MISC) and Native American populations from the Northern region of Brazil (NAT).

SNP ID	Alleles	AFR	AMR	EAS	EUR	SAS	ABraOm	MISC	NAT
rs74597329 *	T	0.364	0.621	0.932	0.683	0.766	0.624	0.744	0.838
	G	0.636	0.379	0.068	0.317	0.234	0.376	0.256	0.162
rs4803221 *	C	0.774	0.721	0.936	0.797	0.845	0.78	0.832	0.937
	G	0.226	0.279	0.064	0.203	0.155	0.22	0.169	0.064
rs73555604	C	0.801	0.977	0.999	0.98	0.984	0.952		
	T	0.199	0.023	0.001	0.02	0.016	0.048		
rs117648444	G	0.932	0.937	0.995	0.906	0.95	0.921		
	A	0.068	0.063	0.005	0.094	0.05	0.079		
rs11322783 *	TT	0.364	0.621	0.932	0.683	0.766	0.624	0.744	0.838
	T-	0.636	0.379	0.068	0.317	0.234	0.376	0.256	0.162

Shaded lines represent the frequencies of major allele and non-shaded represent minor allele. * Represents the SNVs evaluated in native and mixed populations.

**Table 4:** Frequencies of variants of the *IFNL4* gene investigated in African (AFR), American (AMR), East Asian (EAS), European (EUR), South Asian (SAS) populations from the 1000 Genomes Project, Brazilian population from Online Archive of Brazilian Mutations (ABraOm), mixed-race (MISC) and Native Americans from the Northern region of Brazil (NAT).

SNP ID	Alleles	AFR	AMR	EAS	EUR	SAS	ABraOm	MISC	NAT
rs74597329 *	T	0.293	0.597	0.92	0.688	0.758	0.624	0.744	0.838
	G	0.707	0.403	0.08	0.312	0. 242	0.376	0.256	0.162
rs4803221 *	C	0.837	0.7	0.925	0.828	0.834	0.78	0.832	0.937
	G	0.163	0.3	0.075	0.172	0.166	0.22	0.169	0.064
rs73555604	C	0.737	0.976	1	0.983	0.979	0.952		
	T	0.263	0.024		0.017	0.021	0.048		
rs117648444	G	0.925	0.935	0.995	0.882	0.956	0.921		
	A	0.075	0.065	0.005	0.118	0.044	0.079		
rs11322783 *	T-	0.707	0.403	0.081	0.311	0.239	0.376	0.256	0.162
	TT	0.293	0.597	0.919	0.689	0.761	0.624	0.744	0.838

Shaded lines represent the frequencies of major allele and non-shaded represent minor allele. * Represents the SNVs evaluated in native and mixed populations.

**Table 5:** Pairwise comparison (*p*-value) of allelic frequencies in Native, Mixed-population and with each one of the five continental populations from the 1000 Genomes Project database for *IFNL4* gene variants investigated.

	rs74597329	rs4803221	rs11322783
AFR vs. MISC	<0.0001	0.4549	<0.0001
AMR vs. MISC	0.1395	0.1395	0.1395
EAS vs. MISC	0.0031	0.0585	0.0031
EUR vs. MISC	0.4976	0.7734	0.4976
SAS vs. MISC	0.7734	1	0.7734
ABraOm vs. MISC	0.1395	0.5294	0.1395
MISC vs. NAT	0.2281	0.1395	0.2281
AFR vs. NAT	<0.0001	0.0164	<0.0001
AMR vs. NAT	0.0097	0.0031	0.0097
EAS vs. NAT	0.1659	1	0.1659
EUR vs. NAT	0.0638	0.0585	0.0638
SAS vs. NAT	0.4131	0.1395	0.4131
ABraOm vs. NAT	0.0097	0.025	0.0097

Shaded cells represent significant differences between two populations (*p*-value < 0.05).

**Table 6:** Pairwise comparison (*p*-value) of allelic frequencies in Native, Mixed-population and with each one of the five continental populations from GnomAD database for *IFNL4* gene variants investigated.

	rs74597329	rs4803221	rs11322783
AFR vs. MISC	<0.0001	0.4579	<0.0001
AMR vs. MISC	0.1417	0.1417	0.1417
EAS vs. MISC	0.0003	0.057	0.0003
EUR vs. MISC	0.4999	0.7745	0.4999
SAS vs. MISC	0.7745	1	0.7745
ABraOm vs. MISC	0.1417	0.5313	0.1417
MISC vs. NAT	0.2303	0.1417	0.2303
AFR vs. NAT	<0.0001	0.016	<0.0001
AMR vs. NAT	0.0095	0.003	0.0095
EAS vs. NAT	0.1679	1	0.1679
EUR vs. NAT	0.0622	0.057	0.0622
SAS vs. NAT	0.4162	0.1417	0.4162
ABraOm vs. NAT	0.0095	0.0244	0.0095

Shaded cells represent significant differences between two populations (*p*-value < 0.05).

## Data Availability

Data are contained within the article and Supplementary Materials.

## Supplementary Materials

The following supporting information can be downloaded at: https://www.mdpi.com/article/10.3390/genes14112075/s1.
