# Supplementary material for: Variants of IFNL4 Gene in Amazonian and Northern Brazilian Populations
Source: Genes (Basel). 2023 Nov 14;14(11):2075. doi: 10.3390/genes14112075 (PMC10671175; doi:10.3390/genes14112075)

SUPLEMENTARY MATERIAL

Supplementary plot. Differences between populations from GnomAD and 1000 Genomes Project compared to natives and mixed groups of our study.

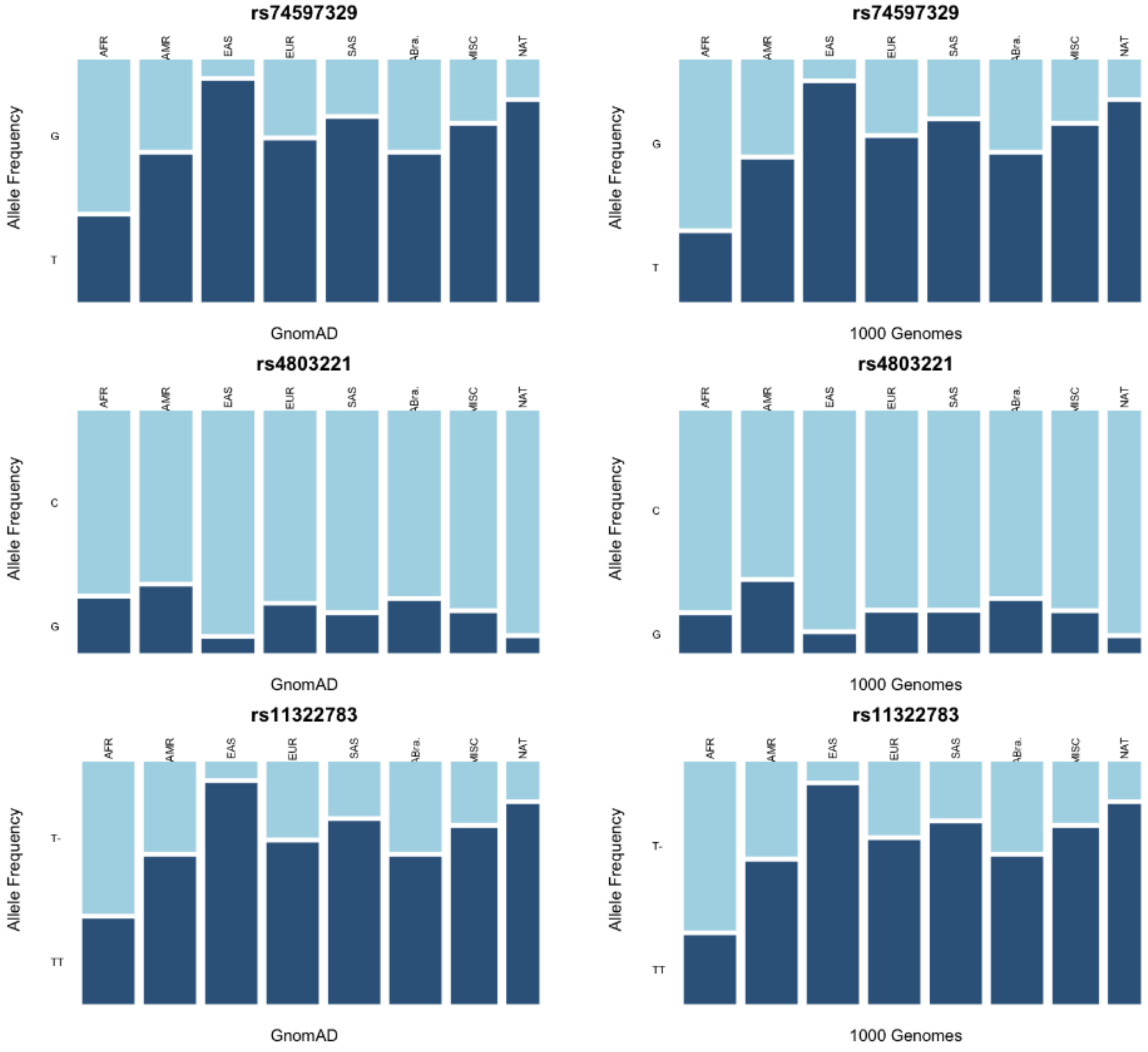

Supplement: Supplementary file 1 [file genes-14-02075-s001.zip › genes-2641426-supplementary.pdf]
